# Impact of Anaemia on HbA1c Interpretation Among Normogylcaemic Individuals With Diabetes Mellitus: A Clinical Laboratory Observational Study

**DOI:** 10.7759/cureus.49901

**Published:** 2023-12-04

**Authors:** Phillip Bwititi, Lexin Wang, Eugene Butkowski, Ezekiel U Nwose

**Affiliations:** 1 Dentistry and Medical Sciences, Charles Sturt University, Wagga Wagga, AUS; 2 Cardiology, Wagga Wagga Medical Centre, Wagga Wagga, AUS; 3 Cardiology, Liaocheng People’s Hospital, Shandong First Medical University, Liaocheng, CHN; 4 Biomedical Sciences, La Trobe University, Albury-Wodonga, AUS; 5 Public and Community Health, Novena University, Amai Campus, Ndokwa West, NGA; 6 Health and Medical Sciences, University of Southern Queensland, Toowoomba, AUS

**Keywords:** lab interpretation, laboratory test, haemoglobin a1c, anaemia, diabetes control

## Abstract

Background and aim: Over the past decades, glycosylated haemoglobin (HbA1c) has been a gold standard for monitoring diabetes control over a long period, relative to blood glucose level (BGL) which measures short-term results. It is speculated that anaemia and factors that induce haemolysis may cause falsely elevated HbA1c leading to ‘false positive’ interpretations. This study aimed to investigate how anaemia impacts HbA1c.

Methods: This was a pathology-based observational pilot study using archived data of diabetic subjects monitored with both BGL and HbA1c in regional New South Wales (NSW), Australia. A total of 28,487 cases of blood glucose results were pooled and those with HbA1c and anaemia results were evaluated for correlation with the BGL results. Data collection was limited to de-identified information from the laboratory information system, hence details on the ethnicity and medical history were unavailable. Descriptive frequencies and Pearson correlations were performed.

Results: In the pooled data, 53.36% of individuals were females, and 50.54% had BGL ≥5.6 mmol/L. In the pilot dataset, the majority (64.86%) were males, 18.92% with BGL ≤5.6 mmol/L and 67.57% had HbA1c (≥6.5%). In the entire dataset, BGL was moderately and positively correlated with HbA1c (r = 0.6), whereas in the subset of individuals with normo-BGL and anaemia, the correlation was negative (r = -0.2).

Discussion: This pilot investigation observed a pertinent issue, which is a negative correlation between glycaemia and HbA1c in patients with anaemia. HbA1c was falsely increased despite normal blood glucose levels in individuals with anaemia. This advances the speculation that anaemia falsely increases HbA1c. Therefore, caution is necessary while interpreting HbA1c results for patients with anaemia, and new methods for interpretation are required.

## Introduction

Glycosylated hemoglobin (HbA1c) has become a gold standard for monitoring diabetic control over the past few decades as it is more informative on glycaemic control over a three-month period by comparison with the frequent blood glucose level (BGL) [1], though questions still persist [2]. The utilization of the HbA1c test has gone beyond determination of diabetes control to include diagnosis of type 2 diabetes mellitus (T2DM) [3-5]. However, there is still some clarity required on the superiority of HbA1c in the screening for diabetes and prediabetes [6].

There are several factors that may influence the accuracy of HbA1c in the diagnosis and monitoring of diabetes [7,8]. Besides comorbidities, other factors include age [9,10], anaemia [11] and genetics [12]. The effect is that HbA1c may not always correlate or match BGL [7], and given the knowledge of positive correlation between BGL and HbA1c [13], the impact is potentially misleading information when interpreting the results of some patients [8]. This misleading information is buoyed by the existence of conversion charts [14], and in research, the impact may also occur if statistical analysis finds a negative correlation between BGL and HbA1c, whereby a researcher with unconscious bias may query data entry, especially if a study is premised on positive correlation.

Some clinical laboratory tests suffer from false negative and positive results for HbA1c, and anaemia is implicated in both false negative and false positive results [8,15]. Thus, there is a conundrum about anaemia in diabetes monitoring. It is also noteworthy that anaemia and hyperbilirubinaemia have been speculated to induce haemolysis, which possibly elevates HbA1c level leading to ‘false positive’ interpretations [16]. Yet, there is a dearth of data to advance this knowledge.

A previous study demonstrated how HbA1c correlates with age in diabetes patients, especially being a weak negative correlation, and that there are probabilities for HbA1c to increase by 1% every two years [17]. How HbA1c correlates with anaemia in diabetes patients has yet to be fully investigated. The pathophysiology of HbA1c related to anaemia [11,16,18,19], which determines potential negative correlation with BGL, requires further clarification and integration in clinical practice [7,19]. Thus, given the implication of anaemia in both false HbA1c results, it is important to elucidate the impact of anaemia on the values of HbA1c and the positive correlation between BGL and HbA1c in order to improve the use of HbA1c in the diagnosis and monitoring of diabetes.

This study aimed to investigate empirical evidence of a negative correlation between BGL and HbA1c and how anaemia impacts such potentially misleading relationship. Further details on these aspects are provided in this study. There have been questions regarding 'When HbA1c and blood glucose do not match: how much is determined by race, genetics and differences in mean red blood cell age?' [7]. Based on an opportunistic observation of negative correction (unpublished), the research question therefore is ‘When HbA1c and blood glucose levels do not match, how is this affected by anaemia?’

The abstract of the study was previously presented at the International Diabetes Federation Congress in 2022 [20].

## Materials and methods

Study design

This was an observational clinical pathology pilot study. It adopted a clinical observational approach because the data are mainly based on observation of clinical results of individual cases being monitored for diabetes control. The study is categorised as clinical pathology-based, as the data were limited to information mined from the laboratory information system of a pathology service, and pilot study as this is an initial relatively small sample study [21].

Ethics approval and considerations

This study was part of a cardiovascular risk assessment in prediabetes and undiagnosed diabetes study [22] and was approved by the Human Research Ethics Committee of Charles Sturt University (#HREC H2014158) in 2014. For ethical reasons, archived data were de-identified [21].

Setting

The data collection was done at the South-West Pathology Service (SWPS) as previously published [21]. The SWPS is an accredited clinical pathology facility of New South Wales (NSW) Health Australia that serves the people living in regional South-Western NSW. High-throughput automated analysers were used in the biochemistry and haematology departments. Beckman Coulter (Mount Waverley: Beckman Coulter Diagnostics) was automated testing equipment in use at the time when test results of this data were generated. Although the objective of this study does not include the investigation of the sensitivity and specificity of past methodologies, suffice that all tests were performed according to standard operational procedures including internal and external quality control compliance.

Population, data and subsample selection

The data comprised information from individuals within a multi-ethnic population who visited the SWPS, South-Western region of New South Wales Australia. De-identified retrospective data of diabetes mellitus cases were mined in a health facility and the data included biochemical and haematological parameters [21]. Among 28,487 diabetes cases from 2006 to 2008, those with the trios’ data of blood glucose level (BGL), haematocrit and HbA1c results were pooled, from which the anaemic pilot data were selected.

Statistical analysis

The statistical analyses were performed using IBM SPSS version 28 (Armonk, NY: IBM Corp.) and Excel analysis tool (Redmond, WA: Microsoft Corp.). Statistical significance was determined at p<0.05. Three statistical analyses were performed. First, a comparison was performed to check if HbA1c differed between dichotomous halves of BGL and/or haematocrit (HCT) in the dataset. Second, to evaluate the possible influence of anaemia in the correlation between the HbA1c and BGL levels, both kurtosis <4.0 and skewness <2.0 levels were used to determine normality in data. Hence Pearson correlations were performed in different data subsets, viz: pilot data, anaemia subset (haematocrit ≤32%, N = 37), anaemia with glucose level <8.0 mmol/L and anaemia with glucose level <5.6 mmol/L [23]. T-test comparison was performed on dichotomous halves of glucose and HCT levels. Third, a critical review for agreement was performed, specifically whether comments on HbA1c results agree with the BGL results. The percentage of disagreement comments was also determined.

## Results

Among the whole dataset (N = 28,487) pooled, a simple majority (53.36%) were females and 14,089 (50.54%) had BGL ≥5.6 mol/L. Less than 33% had the complete triple results of HbA1c, haematocrit and BGL. Of those with complete data, the subset with anaemia (HCT ≤32%) was selected as pilot data (N = 37), which comprised 13 females and a simple majority (64.86%) were males, 18.92% with BGL ≤5.6 mmol/L and 67.57% had HbA1c (≥6.5%). The summary of descriptive statistics of the pilot data is presented in Table 1, which is comparable with the whole dataset.

**Table 1:** Descriptive statistics of pilot data (N = 37). HbA1c: glycosylated haemoglobin

Variables	Mean	Median	SD	Kurtosis	Skewness
Age (years)	75.32	74.00	13.64	4.88	-1.45
HbA1c (%)	7.66	6.90	2.14	3.19	1.73
Haematocrit (%)	29	29	3	3.57	-1.73
Glucose (mmol/L)	10.25	7.30	6.68	2.89	1.67

In ascertaining if HbA1c differs between dichotomous halves of BGL and/or HCT in the dataset, the results show a significant difference in blood glucose (Table 2). Results in the pilot dataset were similar to the whole dataset.

**Table 2:** HbA1c comparison between dichotomous halves of glucose and HCT levels. *N = 28,487. ^**^N = 37. ^***^T-test is used to calculate p-values. HbA1c: glycosylated haemoglobin; HCT: haematocrit; BGL: blood glucose level

Variables	Dichotomy	Whole dataset*	Pilot dataset^**^	p-Value^***^
BGL	Lower 50%	6.15%	6.80%	0.01
	Top 50%	8.31%	8.62%	
HCT	Lower 50%	7.15%	8.12%	0.38
	Top 50%	7.25%	7.29%	

Pearson correlation test results were interpreted according to standard cut-offs [24]. BGL was moderately and positively correlated with HbA1c, including in the pilot data subset with anaemia. However, the correlation changed to negative in those with normoglycaemia or around prediabetic BGL (Table 3). Evaluation of the data inclusive of whether HbA1c levels agree with BGL levels showed that five of the 37 cases had HbA1c results indicative of excellent glycaemic control, but blood glucose was higher than 7.0 mmol/L. Another five of the 37 cases were reported as 'acceptable or very good glycaemic control'; but had glucose levels >11.0 mmol/L. A further five of the 37 cases comprising sequential serial numbers 14-18, excerpt of the 'supplementary information of pilot dataset' (appendix), showed recommendations for change in management for patients with BGL 6.2 and 6.6 mmol/L but excellent glycaemic control and very good diabetes control for patients with BGL 6.3 and 7.0 mmol/L, respectively (Table 4). These sums up to be 15 of the 37 cases, i.e., 40.54% with comments perhaps conflicting with BGL levels.

**Table 3:** Pearson correlations between glucose and HbA1c levels. *Around prediabetic level. HbA1c: glycosylated haemoglobin; BGL: blood glucose level

Variables	N	Correlation
Anaemia	37	0.648118
Anaemic BGL <8.0*	22	-0.11916
Anaemic BGL <5.6	9	-0.18764

**Table 4:** Cases where BGL and HbA1c did not agree. HBA1CT: glycated haemoglobin (HbA1c) test; HBACOM: HbA1c result interpreted comment; GLUC: blood glucose level test; HCT: haematocrit

S.no.	Gender	Age (year)	HBA1CT	HBACOM (unedited and unformatted)	HCT	GLUC
14	F	88	8.6	The HbA1c result indicates poor diabetes control. Recommend change in management.	0.29	6.2
15	F	76	5.8	The HbA1c result, in the presence of diabetes, indicates excellent glycaemic control.	0.255	6.3
16	M	83	8.6	The HbA1c result indicates poor diabetes control.	0.266	6.3
17	M	60	8.4	The HbA1c result indicates poor diabetes control. Recommend change in management.	0.286	6.6
18	M	88	6	The HbA1c result indicates very good diabetes control.	0.272	7.0

## Discussion

This study investigated evidence of the negative correlation between BGL and HbA1c, and how anaemia impacts such relation. The research question arose from 'When HbA1c and blood glucose do not match…' and 'How is the correlation affected by anaemia?' [7]. The assumption was that if haematocrit could induce an increase in HbA1c level, then there would be a tendency for a negative correlation between HbA1c and BGL in anaemia.

The descriptive statistics show that patients were spread across a wide distribution in age, but main variables of interest (BGL, HbA1c and HCT) are normally distributed as indicated by kurtosis <4.0 and skewness <2.0 levels (Table 1). Regarding whether HbA1c differed between dichotomous halves of BGL and/or HCT in the dataset, the results showed that HbA1c level is significantly higher with BGL but not with HCT (Table 2). Another study has reported haematocrit characteristics of participants stratified by HbA1c status and no significant difference is indicated [25].

In the evaluation of correlation between HbA1c and BGL levels, Pearson correlations were positive in the dataset, but negative in the subsets where BGL is normal or near normal (Table 3). The literature shows that haematocrit impacts HbA1c, but there is no empirical data demonstrating a negative correlation. A case study highlighted poor correlation between HbA1c and glycaemia in three renal dialysis patients and attributed the observation to anaemia [19]. There has been concern that anaemia could have a significant impact on the interpretation of HbA1c as a marker of glycaemia monitoring [7,8]. Hence, this report advances an occasion where a negative correlation between HbA1c and BGL can be observed as impacted by anaemia.

Critical review shows that 40.54% of comments were based on HbA1c that conflict with BGL level and this is the crux of the discourse. This observation highlights HbA1c levels probably constituting misleading health information that impacts patients’ management, especially diabetes self-management. This can result in a misleading interpretation. For instance, members of a diabetes self-management support group who have anaemia comorbidity could go for checkup and be given the following advice based on HbA1c level. For someone with BGL 6.2 mmol/L, 'the result indicates very poor diabetes control and strongly recommend change in management!' For another with BGL 7.0 mmol/L, 'the result indicates very good diabetes control!'

Such misleading information will confuse the patients. Even if the clinician tries to explain the supremacy of HbA1c over BGL but without recourse to the coexisting anaemia, patients will likely query the need for BGL monitoring. This confusion is supported by the knowledge that fasting BGL may be more accurate than HbA1c in general population screening, especially when factors, such as age, gender and obesity, are considered [26]. Hence, this study advances careful cognizance of potential negative correlation due to anaemia in occasions of discordant HbA1c versus BGL levels that may mislead clinical advice with implications on diabetes management.

For brevity, this report does not discount the value of HbA1c. There is no arguing the fact about the relationship between HbA1c and BGL, and the results in Table 2 confirm that HbA1c level is statistically significantly higher in the upper 50% of BGL range compared to the lower half. However, it has been advised that there are occasions when HbA1c results may be misleading [8]. Hence, many factors affecting its relationship with BGL may limit its applicability to all diabetes populations [13]. What this report contributes is an empirical elucidation of occasions where a blanket application of the relationship constitutes misleading health information.

The implication for clinical research and practice is the need to include haematocrit assessment in diabetes monitoring, especially when HbA1c and blood glucose do not match. In clinical practice, a good proportion of patients do go for the HbA1c test alone without BGL and/or haematocrit. The suggestion being put forward is to check that BGL matches high HbA1c, and if not, haematocrit should be checked. In research involving the assessment of HbA1c, it is quite commonplace that haematocrit level of participants gets overlooked [27]. Thus, it is worthy to recommend that practitioners and researchers review their protocols and interpret HbA1c results with a consciousness of when HbA1c and blood glucose do not match and factors that interfere with the relationship. It is sufficient to note that the interest in continuing with routine blood glucose tests and possibly exploring better ways of utilizing the test result has remained warranted [28].

Limitation and scope of this study

This study is limited to archived clinical pathology laboratory data, only. The details on the ethnicity, medical history and diabetes status of the subjects are lacking. However, the missing details are neither expected nor supposed to impact the clinical interpretation of HbA1c results on individual person-centered care. In the same vein, information on other medical conditions/drugs associated with these patients that can affect red blood cell turnover is out-of-scope of this laboratory data. It has been speculated that inflammation may constitute interference in HbA1c pathophysiology, though not after adjusting for age, gender and metabolic syndrome indices [29]. Therefore, non-investigation of inflammatory markers is hereby acknowledged as another limitation in this study. Furthermore, this study has focused on 'the degree of the relationship' not on the prediction of response, hence statistical analysis of relation was limited to correlation and not regression.

## Conclusions

This pilot study used observational clinical pathology data to investigate the fundamental issue of 'when HbA1c and blood glucose do not match.' The observations presented in this study reveal two stepwise issues albeit advance what has been speculated. Firstly, a possible negative correlation between glycaemia and HbA1c among the subset of individuals living with diabetes. Secondly, the novel aspect of this contribution may lie in the observation that the negative correlation observed is mainly in those maintaining BGL within the reference range. Data show that HbA1c was falsely increased despite glycaemic control in the subset of patients with anaemia comorbidity, thus advancing the speculation that anaemia may falsely increase HbA1c. Therefore, clinical interpretations of HbA1c results should take cognizance of both anaemia status and blood glucose levels.

## Appendices

**Table 5:** Supplementary information - pilot dataset. HBA1CT: glycated haemoglobin (HbA1c) test; HBACOM: HbA1c result interpreted comment; GLUC: blood glucose level test; HCT: haematocrit

S. no.	Gender	Age (years)	HBA1CT	HBACOM (unedited and unformatted)	HCT	GLUC
1	M	71	6.7	The HbA1c result indicates very good diabetes control.	0.297	3.1
2	M	76	7.3	The HbA1c result indicates acceptable diabetes control.	0.289	4.3
3	M	23	5.9	The HbA1c result, in the presence of diabetes, indicates excellent glycaemic control.	0.299	4.6
4	M	74	6.7	The HbA1c result indicates very good diabetes control.	0.18	4.8
5	M	93	6.8	The HbA1c result indicates very good diabetes control.	0.304	5.1
6	M	87	7.1	The HbA1c result indicates acceptable diabetes control.	0.283	5.3
7	F	69	6	The HbA1c result indicates very good diabetes control.	0.293	5.4
8	F	84	6.9	The HbA1c result indicates very good diabetes control.	0.264	5.5
9	M	75	6.6	The HbA1c result indicates very good diabetes control.	0.295	5.5
10	M	91	6.2	The HbA1c result indicates very good diabetes control.	0.277	5.7
11	F	74	6.8	The HbA1c result indicates very good diabetes control.	0.219	5.8
12	M	67	6.1	The HbA1c result indicates very good diabetes control.	0.254	6.1
13	F	69	5.9	The HbA1c result, in the presence of diabetes, indicates excellent glycaemic control.	0.309	6.1
14	F	88	8.6	The HbA1c result indicates poor diabetes control. Recommend change in management.	0.29	6.2
15	F	76	5.8	The HbA1c result, in the presence of diabetes, indicates excellent glycaemic control.	0.255	6.3
16	M	83	8.6	The HbA1c result indicates poor diabetes control.	0.266	6.3
17	M	60	8.4	The HbA1c result indicates poor diabetes control. Recommend change in management.	0.286	6.6
18	M	88	6	The HbA1c result indicates very good diabetes control.	0.272	7
19	M	71	5.8	The HbA1c result, in the presence of diabetes, indicates excellent glycaemic control.	0.313	7.3
20	F	98	5.9	The HbA1c result, in the presence of diabetes, indicates excellent glycaemic control.	0.294	7.7
21	F	66	5.5	The HbA1c result, in the presence of diabetes, indicates excellent glycaemic control.	0.317	7.7
22	F	71	8.1	The HbA1c result indicates poor diabetes control.	0.316	7.8
23	M	66	6.1	The HbA1c result indicates very good diabetes control.	0.308	10.8
24	M	85	9.2	The HbA1c result indicates very poor diabetes control. Strongly recommend change in management.	0.317	10.9
25	M	73	6.5	The HbA1c result indicates very good diabetes control.	0.305	12.1
26	M	91	5.7	The HbA1c result, in the presence of diabetes, indicates excellent glycaemic control.	0.296	12.2
27	M	81	7.6	The HbA1c result indicates acceptable diabetes control.	0.31	12.3
28	M	74	9.8	The HbA1c result indicates very poor diabetes control. Strongly recommend change in management.	0.314	12.5
29	M	74	14.9	The HbA1c result indicates very poor diabetes control.	0.232	12.8
30	M	94	7.1	The HbA1c result indicates acceptable diabetes control.	0.315	13.9
31	F	72	7.5	The HbA1c result indicates acceptable diabetes control.	0.302	14.9
32	F	70	7.5	The HbA1c result indicates acceptable diabetes control.	0.312	16.9
33	M	55	11.9	The HbA1c result indicates very poor diabetes control. Strongly recommend change in management.	0.286	19
34	M	76	9.8	The HbA1c result indicates very poor diabetes control.	0.285	19.9
35	M	82	9.4	The HbA1c result indicates very poor diabetes control. Strongly recommend change in management.	0.25	23
36	F	84	9.6	The HbA1c result indicates very poor diabetes control.	0.291	24.5
37	F	56	13	The HbA1c result indicates very poor diabetes control.	0.295	33.2
